# Declining hepatitis A seroprevalence in adults in Catalonia (Spain): a population-based study

**DOI:** 10.1186/1471-2334-7-73

**Published:** 2007-07-04

**Authors:** Àngela Domínguez, Miquel Bruguera, Pere Plans, Jordi Espuñes, Josep Costa, Antoni Plasencia, Lluis Salleras

**Affiliations:** 1 Directorate of Public Health. Generalitat of Catalonia. Travessera de les Corts, 131-159, 08028 Barcelona, Spain; 2 Department Public Health. University of Barcelona. C. Casanovas, 143, 08036 Barcelona, Spain; 3 Liver Unit. Hospital Clínic. C. Villaroel, 170, 08036 Barcelona, Spain; 4 Service of Microbiology, Hospital Clínic. C. Villaroel, 170, 08036 Barcelona, Spain

## Abstract

**Background:**

One of the main uses of seroprevalence studies it to evaluate vaccination programmes. In 1998, a programme of universal vaccination of preadolescents in schools with the hepatitis A vaccine was begun in Catalonia. The objective of this study was to investigate the prevalence and risk factors of hepatitis A virus infection (HAV) in a sample of the adult population of Catalonia in 2002 and to evaluate the changes with respect to a survey carried out in 1996.

**Methods:**

The prevalence of HAV antibodies was determined by a third generation competitive immunometric assay in a representative sample of 1292 people aged >15 years. The association between the prevalence and different sociodemographic variables was determined by multiple logistic regression analysis.

**Results:**

The standardized global prevalence of HAV antibodies in 2002 was 68.2%, increased with age (p < 0.0001) and was associated with being born outside Catalonia (OR: 1.75; 95% CI 1.11–2.76) and lower social class (OR: 1.14; 95% CI 1.05–1.25). Compared with the last survey carried out in 1996 the standardized global prevalence was lower (68.2% vs 77.8%; p < 0.0001) as was the prevalence in people under 45 years.

**Conclusion:**

The prevalence of the hepatitis A virus is decreasing in the adult population of Catalonia, especially in the younger age groups. The programme of vaccination of adolescents begun in 1998 to control the disease can provide indirect protection to the unvaccinated population.

## Background

Hepatitis A virus infection (HAV) has decreased substantially in developed countries in recent decades, due to improvements in the quality of the water supply and the treatment of human residual wastes [1]. However, in these countries, the rates of clinical disease may increase, since the virus circulates less, infections in childhood (which are usually asymptomatic) are less frequent, and infections in adults, which are more likely to have clinical symptoms, increase. Although most cases are benign, the disease may present relapses or a prolonged course and produce extrahepatic complications, fulminant hepatitis or even death [2-4].

The availability of a hepatitis A vaccine since the middle of the 1990s, with a high degree of efficacy [5,6] and effectiveness [7,8] has increased discussion on the vaccination strategy to a disease with an exclusively human reservoir and which is therefore potentially eradicable [9].

Although current strategies are centred on the control and not the eradication of the disease [10], it is important to determine the pattern of hepatitis A virus infection in each community in order to optimize vaccination strategies.

In Catalonia, the hepatitis A vaccine was introduced in 1995 and was recommended for persons belonging to risk groups. At the end of 1998, a pilot programme of vaccination of preadolescents at 12 years of age in schools with the combined hepatitis A+B vaccine was begun, and every school year a coverage > 90% was reached[11].

Seroprevalence studies are recognized to be useful tools to evaluate the impact of vaccination programmes [12].

The objective of this study was to determine the prevalence of hepatitis A antibodies in the adult population of Catalonia and to compare the results with those obtained in the surveys carried out in 1989 and 1996 using a similar methodology [13,14].

## Methods

The study was carried out in 2002. The population sample was obtained in a two-stage process. Firstly, 97 municipalities were selected randomly after stratification into urban (>10,000 inhabitants) and rural (≤10,000 inhabitants) habitat. Secondly, individuals to be included in the study were selected randomly from the municipal census lists. Written informed consent was sought from all participants. The study was approved by the Bioethics Committee of the Department of Health.

The sample size was calculated using an alpha error of 5% and a precision of ± 0.025, with an expected prevalence of antibodies of 50% (the least favourable situation), corresponding to a theoretical sample size of 1,600 people.

Sera samples were kept frozen at -40°C until analysis in the microbiology laboratory of the Hospital Clinic of Barcelona.

Total HAV antibodies were determined by a commercial third-generation competitive immunometric assay (ETI-AB-HAVK-3; DiaSorin, Saluggia [Vercelli], Italia) according to the manufacturer's instructions.

Sociodemographic variables were collected by a questionnaire that included age, sex, social class, municipality of residence and place of birth. The municipality of residence was classified as rural (<10,000 inhabitants) or urban (≥10,000 inhabitants). Social class was determined using the occupation (or the occupation of the parents in schoolchildren) according to the English classification [15]. Age and sex standardized prevalences for 1989, 1996 and 2002 were calculated using the Catalan population of 2001 as the standard population. Thus, the global prevalence was obtained by weighting the prevalence from different age group and taking into account the distribution of the Catalan population in 2001 [16].

The standardized global age and sex prevalence and the age specific prevalences of HAV antibodies were compared with the prevalences obtained in the 1989 and 1996 studies in representative samples of the Catalan population using the same methodology [13,14]. The differences between proportions were compared using the Chi-square and Fisher tests. The level of statistical significance was established at p < 0.05.

The Odds Ratio and the 95% confidence intervals (CI) were calculated for the sociodemographic variables. A multiple logistic regression analysis was carried out including age, sex and the variables that were significant in the bivariate analysis.

The statistical analysis was performed using the SPSS for programme Windows version 10 (SPSS Inc, Chicago, Ill, USA).

## Results

The final sample obtained was 1292 persons, a global participation of 81%. The distribution of the sample was similar to that of the Catalan population. The standardized global prevalence of hepatitis A antibodies was 68.2%, lower than the standardized prevalence of 77.8% obtained in the 1996 study; the difference was statistically significant (p < 0.0001). The differences observed between the standardized global prevalence of the 1989 (84.3%) and 1996 (77.8%) studies were also statistically significant (p = 0.002).

The distribution of the seroprevalences obtained in 1989, 1996 and 2002 according to age group is shown table 1. In the three studies, the prevalence of antibodies increased with age (p < 0.0001). In 2002, the 15–24 years, 25–34 years and 35–44 years age groups had prevalences of 15.4%, 34.9% and 75.1%, respectively, all inferior to the corresponding age groups in the 1996 study (31.4%, 56.9% and 86.9%); the differences were statistically significant (p = 0.004, p < 0.0001 and p = 0.002, respectively).

In the univariate analysis of the sociodemographic variables, significant differences were detected for the place of birth (OR: 4.57; 3.15–6.66) and social class (OR: 1.72; 1.31–2.27) (table 2). These associations were maintained in the multivariate analysis, with adjusted OR of 1.75 (1.11–2.76) and 1.14 (1.05–1.25), respectively.

**Table 1:** Prevalence of HAV antibodies according to age groups. Catalonia, 1989, 1996 and 2002.

Age groups	1989 Prevalence			1996 Prevalence			2002 Prevalence		
						
	%	(95%CI)	n	%	(95%CI)	n	%	(95% CI)	n
15–24	43.1	(31.4–55.3)	72	31.4^2^	(23.1–40.5)	118	15.4^2^	(9.8–22.6)	136
25–34	82.1^1^	(72.3–89.6)	84	56.9^1,3^	(50.1–63.6)	216	34.9^3^	(28.7–41.6)	223
35–44	93.8	(88.1–97.3)	129	86.9^4^	(81.8–91.1)	222	75.1^4^	(69.4–80.1)	265
45–54	95.4	(89.5–98.5)	108	95.5	(91.9–97.8)	222	93.8	(90.3–96.4)	276
55–64	91.0	(82.4–96.3)	78	99.1	(96.8–99.9)	224	97.3	(94.3–99.0)	224
>64	96.1	(90.3–98.9)	102	98.8	(96.6–99.8)	257	98.2	(94.9–99.6)	168
TOTAL*	84.3^4^	(81.3–87.3)	573	77.8 ^4,1^	(75.5–80.1)	1259	68.2^1^	(65.7–70.7)	1292

^1^p < 0.0001 ^2^p = 0.004 ^3^p < 0.0001 ^4^p = 0.002

* Standardized by age and sex

**Table 2:** Sociodemographic variables associated with the prevalence of HAVantibodies in adults.

Variables		Prevalence				
					
		%	(95%CI)	n	OR (95%CI)	ORaj (95%CI)
Sex	male	73.4	(69.8–77.0)	572	1.06 (0.83–1.36)	
	female	72.2	(68.9–75.5)	720	1	
Birthplace	Catalonia	65.5	(62.4–68.6)	890	1	1
	Other place	89.7	(86.6–92.8)	378	4.57 (3.15–6.66)^1^	1.75 (1.11–2.76)^2^
Habitat	urban	71.9	(69.2–74.6)	1070	1	
	rural	77.0	(71.0–83.0)	222	1.31 (0.93–1.84)	
Social class	I-III	66.7	(62.5–70.9)	492	1	1
	IV-V	77.5	(74.2–80.8)	618	1.72 (1.31–2.27)^1^	1.14 (1.05–1.25)^3^

^1^p < 0.0001; ^2^p = 0.0160; ^3^p = 0.002

CI: Confidence interval

ORaj = Odds Ratio adjusted by multiple logistic regression analysis for age, sex, birthplace and social class.

## Discussion

The standardized prevalence of hepatitis A virus infection in the adult population of Catalonia decreased globally by 10% during the years 1996–2002, while the reduction during the period 1989–1996 was only 8%. In the 1996–2002 period, the decrease was greatest in the youngest age groups: 51% in the 15–24 years age group, 39% in the 25–34 years age group and 14% in the 35–44 years age group.

In the 1996 study, at a time when vaccination was only recommended for risk groups, there was a significant reduction (31%) with respect to the 1989 study in the 25–34 years age group but not in the other two groups [13,14].

It is clear that improvements in hygiene and cleanliness are determining factors of the prevalence of HAV infection in the community. However it is not so clear why such improvements have a higher impact in the adult population in the 1996–2002 period than in the 1989–1996 period. In our opinion it is probable that the vaccination programme of preadolescents begun in 1998 have got some influence in this reduction.

Both observational studies [7,17] and mathematical models [12] carried out in the United States in areas where routine vaccination is recommended, suggest the hepatitis A vaccine can not only prevent infection in the vaccinated population but also provide indirect protection to the unvaccinated population. In Israel, where universal vaccination with two doses of vaccine at 18 and 24 months of age was introduced in 1999, and where coverage was 90% for the first dose and 85% for the second, a high degree of herd immunity has been observed [18].

In Catalonia, the coverage of the routine vaccination programme of preadolescents with the hepatitis A+B vaccine is greater than 90% [19] and when the present study was carried out four cohorts had already been vaccinated. Thus it is plausible to suggest that the indirect effect of the vaccination could explain at least in part, the reduction in the prevalence of the infection observed in adults between 1996 and 2002.

The fact that the greatest reductions in prevalence occurred in young people and young adults might be because these age groups have more contact with the vaccinated population and therefore benefit more from the reduction in the circulation of the virus as a consequence of vaccination.

The fact that the prevalence has not decreased in older people suggests a cohort effect. This may be because older people had a greater probability of becoming infected due to poorer hygiene and sanitation in the past [20].

The available data on disease incidence also corroborate the benefits of universal vaccination. Thus, the reported incidence of the disease in Catalonia in 1996–1998 was 6.2 per 100,000 globally and 10.3 for the 10–14 years age group, whereas after the introduction of universal vaccination, the respective figures for 1999–2001 were 2.6 and 1.8 per 100,000 [11]. However, because hepatitis A incidence shows cyclic increases and decreases, determining how much of the declines observed has resulted from vaccination is complicated [21]; besides, in population-based studies we don't have the benefit of a control community [7].

Of the sociodemographic variables, those associated with a greater prevalence of HAV antibodies were being born outside Catalonia and belonging to the lower social classes. Migration normally involves a flow from less-developed rural nuclei to urban nuclei and it is probable that this explains the association found [22,23].

Likewise, the association between the prevalence of antibodies and the lower socioeconomic level also coincides with the results of other studies [4,24,26].

Taking into account the difficulty of obtaining high vaccination coverages in those population groups with a greater risk of infection and also the fact that vaccination coverages are very high in Catalonia, the strategy of universal vaccination is, in our opinion, the best option. The greatest drawback of routine vaccination is the cost of the vaccine [10]. The hepatitis A+B vaccination programme implemented in Catalonia, with the substitution of the hepatitis B vaccine by the A+B vaccine, has been shown to be highly efficient [27].

## Conclusion

The results of this study, carried out four years after the introduction of a pilot programme of vaccination of preadolescents with the hepatitis A+B vaccine in Catalonia in 1998, indicates that the prevalence of HVA has decreased in the adult population, especially in the younger age groups and suggest that vaccination should continue, as probably it has contributed to reducing the circulation of the virus in young adults and to a smaller risk of becoming infected.

Continuation of routine vaccination is also supported by the fact that the reduction in the prevalence of HAV infection in the adult population in Catalonia, especially in the younger age groups, will favour both a shift in clinical cases of the infection to older age groups and the appearance of outbreaks [10,28-30]. This phenomenon has been observed in other southern European countries [30,31] as also in Catalonia [33].

## Competing interests

The author(s) declare that they have no competing interests.

## Authors' contributions

AD and PP designed and coordinate the study. JC performed the immunomedric assay. JE collected sociodemographic data and epidemiological data. MB, LS and AP analysed and discussed the associations obtained. All authors partaicipated in the data analysis. AD and MB drafted the manuscript. All authors read and approved the final version of the manuscript.

## Note

Table 1. Prevalence of HAV antibodies according to age grups. Catalonia 1989, 1996 and 2002.

Table 2. Sociodemographic variables associated with the prevalence of HAV antibodies in adults.

## Pre-publication history

The pre-publication history for this paper can be accessed here:


